# Rapid proteomic analysis for solid tumors reveals LSD1 as a drug target in an end‐stage cancer patient

**DOI:** 10.1002/1878-0261.12326

**Published:** 2018-06-14

**Authors:** Sophia Doll, Maximilian C. Kriegmair, Alberto Santos, Michael Wierer, Fabian Coscia, Helen Michele Neil, Stefan Porubsky, Philipp E. Geyer, Andreas Mund, Philipp Nuhn, Matthias Mann

**Affiliations:** ^1^ Department of Proteomics and Signal Transduction Max Planck Institute of Biochemistry Martinsried Germany; ^2^ Novo Nordisk Foundation Center for Protein Research Faculty of Health Sciences University of Copenhagen Denmark; ^3^ Department of Urology University Medical Centre Mannheim University of Heidelberg Mannheim Germany; ^4^ Department of Pathology University Medical Centre Mannheim University of Heidelberg Mannheim Germany

**Keywords:** case study, clinical proteomics, epigenetics, mass spectrometry, urachal carcinoma

## Abstract

Recent advances in mass spectrometry (MS)‐based technologies are now set to transform translational cancer proteomics from an idea to a practice. Here, we present a robust proteomic workflow for the analysis of clinically relevant human cancer tissues that allows quantitation of thousands of tumor proteins in several hours of measuring time and a total turnaround of a few days. We applied it to a chemorefractory metastatic case of the extremely rare urachal carcinoma. Quantitative comparison of lung metastases and surrounding tissue revealed several significantly upregulated proteins, among them lysine‐specific histone demethylase 1 (LSD1/KDM1A). LSD1 is an epigenetic regulator and the target of active development efforts in oncology. Thus, clinical cancer proteomics can rapidly and efficiently identify actionable therapeutic options. While currently described for a single case study, we envision that it can be applied broadly to other patients in a similar condition.

**Abbreviations**
ACACB: acetyl‐CoA carboxylase 2
ACOT2: acyl‐coenzyme A thioesterase 2
AIFM2: apoptosis‐inducing factor 2
CGI: cancer genome interpreter
COL11A1: collagen alpha‐1 (XI) chain
CRP: c‐reactive protein
EGFR: epidermal growth factor receptor
FDR: false discovery rate
FFPE: formalin‐fixed paraffin‐embedded
FOLFOX: folinic acid, fluorouracil, and oxaliplatin
GSEA: gene set enrichment analysis
HPLC: high‐performance liquid chromatography
iST: inStageTip
LFQ: label‐free quantification
LSD1/KDM1A: lysine‐specific histone demethylase 1
MAO: monoamine oxidase
MRI1: methylthioribose‐1‐phosphate isomerase
MS: mass spectrometry
PC: pyruvate carboxylase
RSU1: ras suppressor protein 1
SLC22A18: solute carrier family 22 member 18
THBS2: thrombospondin‐2
XELOX: oxaliplatin and capecitabine

## 1 Introduction

Genomic and transcriptomic investigations based on next‐generation sequencing have revolutionized the field of oncology in the last decade and allowed the molecular profiling of thousands of tumors in different cancer types (Cancer Genome Atlas Research Network *et al*., 2013; Stratton *et al*., 2009). While these technologies have led to a better understanding of cancer origin and heterogeneity, it has often been challenging to turn mutation patterns into actionable therapeutic suggestions. It has also become evident that the development and complexity of cancer cannot be understood at the genetic or transcriptomic level alone. Clearly, proteins, the driving biological entities in cells, also play crucial roles in cancer. So far, proteomics—the large‐scale study of all proteins in a given system—has lagged behind genomics for technological reasons. However, following groundbreaking advances in mass spectrometry (MS)‐based proteomics, comprehensive characterization of nearly complete proteomes has now become a reality (Aebersold and Mann, 2016; Bekker‐Jensen *et al*., 2017). In parallel, several proteomic tumor analysis consortia (e.g. CPTAC) have been launched and aim to systematically identify and characterize cancer‐relevant proteins. So far, these consortia have focused on knowledge generation, rather than on specific clinical applications.

Here, we set out to use state‐of‐the‐art proteomics technology directly in a clinical oncology context, as the main focus of this work. Our group has already established proteomic workflows enabling processing of clinically relevant tissue samples to great depth, even for formalin‐fixed paraffin‐embedded (FFPE) material (Wiśniewski *et al*., 2011, 2013). Recently, we have combined nearly all sample processing steps in a single reaction tube, thereby reducing preparation time, contamination, and loss, while increasing quantification accuracy in tissues (inStageTip or iST method) (Kulak *et al*., 2014; Doll *et al*., 2017). We reasoned that these advances would now enable rapid analysis of individual tumor tissues to inform treatment decisions, especially in patients with rare and end‐stage cancer, where evidence for therapeutic strategies and clinical trials are often lacking.

Urachal carcinomas originate from a remnant of the fetal structure connecting the allantois and the bladder. This form of cancer is rare, accounting for less than 1% of all bladder cancers, aggressive, and consequently little studied. Patients with metastatic urachal cancer have very poor prognosis, with median survival of about 1.3 years. Currently, there are no standard chemotherapeutic regimens for metastatic urachal carcinomas, as prospective clinical trials are hampered by its rarity (Molina *et al*., 2007; Szarvas *et al*., 2016). Only a few cases have been investigated at the genomic level (Collazo‐Lorduy *et al*., 2016; Singh *et al*., 2016), and there are no global protein expression profiles of urachal carcinoma that could support the search for biomarkers, therapeutic targets, or disease signatures.

A 57‐year‐old woman presented with a progressive pulmonary metastasis of a recurrent metastatic urachal carcinoma after multimodality treatment including surgery, chemotherapy regimens, and radiation therapy. The patient wished to continue treatment with no further systemic treatments currently available. Based on the iST sample preparation method, we developed a workflow capable of producing analysis results of clinical cancer tissues in only about two days. Profiling the proteomic landscape of the metastasized tumor in comparison with the normal appearing surrounding tissue, we aimed to uncover potential therapeutic targets and gain a deeper understanding of the molecular mechanisms underlying this disease and its progression. We also employed proteomics to characterize the archived primary tumor and compared our results to deep sequencing data that we obtained from the same metastases.

## 2 Results

### 2.1 Prior clinical course

**Figure 1:**
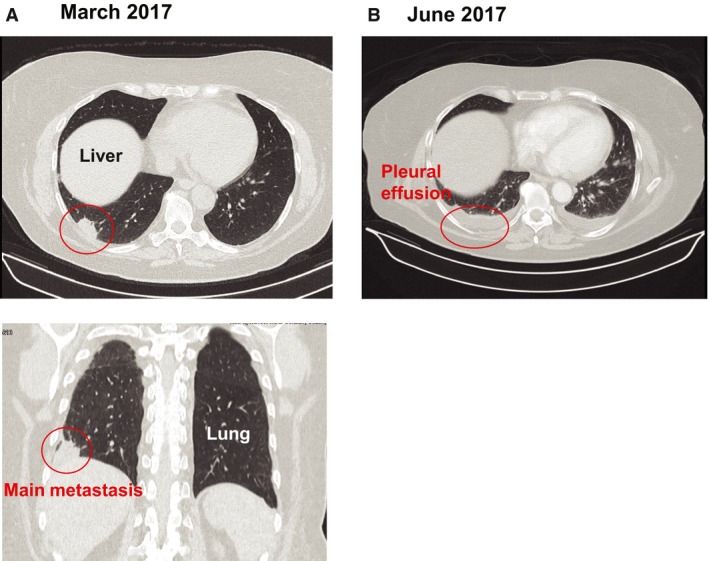
Preoperative CT scans of the urachal carcinoma patient. (A) CT scan in March 2017 showing a metastasis in the right lung. (B) CT scan in June 2017 depicting a pleural effusion before the surgery hiding a pleural carcinosis

Initially, our patient was referred to the Department of Urology at the University Medical Center Mannheim in 2013. Early symptoms included gross hematuria, which led us to perform a subsequent cystoscopy and bladder biopsy. Histopathology revealed a mucinous adenocarcinoma in the bladder, a finding consistent with a diagnosis of urachal carcinoma. As a first line of treatment, we performed a partial cystectomy and lymphadenectomy. Our final pathology showed a pT3b, pN1, L1, V1, R0 mucinous urachal carcinoma of the bladder (Fig. S1A). Follow‐up CT scans were performed on a 3‐month basis. Nine months after resection, the CT scan revealed two suspicious hypodense lesions in the liver (segments 5 and 4a) as well as a local recurrence found at the bladder dome (Fig. S1B,C). The local tumor board recommended chemotherapy, including one cycle of XELOX (oxaliplatin and capecitabine) and nine cycles of FOLFOX (folinic acid, fluorouracil, and oxaliplatin). Chemotherapy led to a partial hepatic response but was stopped due to severe peripheral neuropathy. To assess further treatment strategies, the local recurrence was biopsied and confirmed transurethrally. After tumor board consultation, we performed a resection of the local recurrence combined with a partial hepatectomy and subsequent radiotherapy of the local recurrence side (59.4 Gy). In later stages, two metastases were diagnosed at the introitus vaginae and the CT scan of the thorax revealed bilateral noduli. Subsequent chemotherapy with four cycles of gemcitabine/cisplatin led to a mixed response, and further pulmonary progression of a predominant singular nodule was diagnosed (Fig. 1A). At this point, all standard treatment options were exhausted and we set out to resect the lung metastasis and surrounding healthy tissue for subsequent proteomic analyses. Due to medical and psychological issues, the resection was delayed for two months. In the thoracoscopy, a disseminated pleural carcinosis was observed that was most likely covered by pleural effusion in the preoperative CT scan (Fig. 1B). Pleural metastases and healthy pleura were biopsied, washed in PBS, flash‐frozen, and immediately transferred for proteomic analyses within 1 day.

### 2.2 Streamlined proteomics workflow applied to chemorefractory carcinoma

To be useful in a clinical oncology setting, we reasoned that any proteomic workflow would need to fulfill several criteria, including rapid overall analysis time (few days), extreme sensitivity (few thousand cells), depth of quantitative proteome coverage (several thousand proteins) along with robustness and reproducibility. The workflow that we adapted fulfills all these criteria (see Methods section for details): Briefly, we performed all sample preparation in a single reaction vial, based on the iST sample preparation method (Kulak *et al*., 2014). We chose a single‐run LC‐MS/MS workflow, rather than prefractionating the sample, to minimize measurement time and maximize quantitative accuracy. All bioinformatic analysis was done in the freely available maxquant and perseus software environments (Cox and Mann, 2008; Tyanova *et al*., 2016).

**Figure 2:**
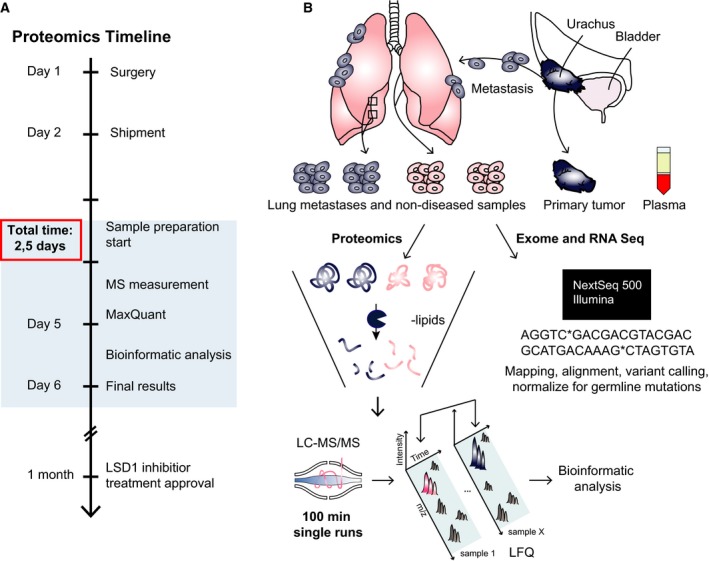
Proteomics workflow for the case study. (A) Timeline of the project. (B) Experimental design, including source of material, inStageTip sample preparation, and depiction of the analytical workflow.

Upon receipt of the samples in the late afternoon, we started by lysing the tissues and extracting the proteins. The surrounding fat was removed by high‐speed centrifugation. Proteins were subsequently digested overnight using specific proteases. On the following day, we analyzed the peptide mixtures using a state‐of‐the‐art label‐free workflow on a quadrupole—Orbitrap mass spectrometer (Fig. 2B). Each sample, constituting a few microgram of material, was measured using 100‐min high‐performance liquid chromatography (HPLC) gradients. Analysis in MaxQuant specified a false discovery rate (FDR) of less than 1% at the peptide and protein levels. In total, we identified 50 870 sequence‐unique peptides, corresponding to 5562 protein groups (proteins that can be distinguished based on the available peptide information). The MaxLFQ algorithm (Cox *et al*., 2014) quantified 5543 proteins in total, with similar coverage in all samples. For further analysis, we only considered the subset of 4857 proteins in our data with quantitative valid values across at least 70% of the samples. Mean sequence coverage of all proteins by identified peptides was about 25%. Signal intensities for the quantified proteins spanned about five orders of magnitudes, with hemoglobin as one of the most abundant proteins, despite extensive washing of the samples with PBS before sample processing. Quantitative reproducibility between technical replicates (same tissue origin but independent analysis workflow) was excellent, demonstrated by Pearson correlation coefficients between 0.97 and 0.99 that are similar to previously achieved values in cell line systems (Coscia *et al*., 2016). We likewise observed high correlation values between control tissues taken from different locations (0.92) and between two different samplings of the metastases (0.97). The complete workflow can be performed in less than 2.5 days, which is an important advantage for application in the clinic.

### 2.3 Proteome analysis reveals LSD1 as a potential therapeutic target

For a functional view of the proteomic data, we used volcano plots to compare expression differences between lung pleural metastases and healthy‐appearing pleura. Based on a t‐test for binary comparison and employing a 5% FDR, we found that 108 (2.2%) proteins showed significant alteration, of which 47 were up‐ and 61 downregulated in the metastases. Gene set enrichment analysis (GSEA) using gene set collections from the MSigDB (Subramanian *et al*., 2005) revealed that proteins upregulated in the metastases were significantly enriched for the terms epithelial‐mesenchymal transition, tumor invasiveness, and tumor metastasis (*P* < 5 × 10^−6^). For example, periostin (POSTN), which has previously been reported to promote cell motility in several cancer types, was 13‐fold higher expressed in the metastases compared to nondiseased tissue (Gillan *et al*., 2002; Ishiba *et al*., 2014; Mikheev *et al*., 2015). The most upregulated (>100‐fold) protein in the metastases was thrombospondin‐2 (THBS2) and is also involved in cell invasion as well as angiogenesis and correlates with poor survival (Bornstein, 2009; Iruela‐Arispe *et al*., 2004; Lin *et al*., 2016; Qian *et al*., 2017; Wang *et al*., 2016). Another protein driving cell invasion, methylthioribose‐1‐phosphate isomerase (MRI1) was highly significantly upregulated but only 1.9‐fold (Kabuyama *et al*., 2009). These observations demonstrate that the proteomic experiment performed as expected and suggest an important role of some of the regulated proteins in the metastatic progression of urachal carcinoma.

In contrast, downregulated proteins were significantly enriched in mitochondrial proteins (*P* < 10^−17^), such as pyruvate carboxylase (PC), acetyl‐CoA carboxylase 2 (ACACB), and acyl‐coenzyme A thioesterase 2 (ACOT2). Interestingly, Ras suppressor protein 1 (RSU1) was about fourfold lower expressed in the metastases. Apoptosis‐inducing factor 2 (AIFM2) was 28‐fold downregulated in the metastases compared with nondiseased tissue. This suggests a regulatory role of RSU1 and AIFM2 in urachal carcinoma metastases.

**Figure 3:**
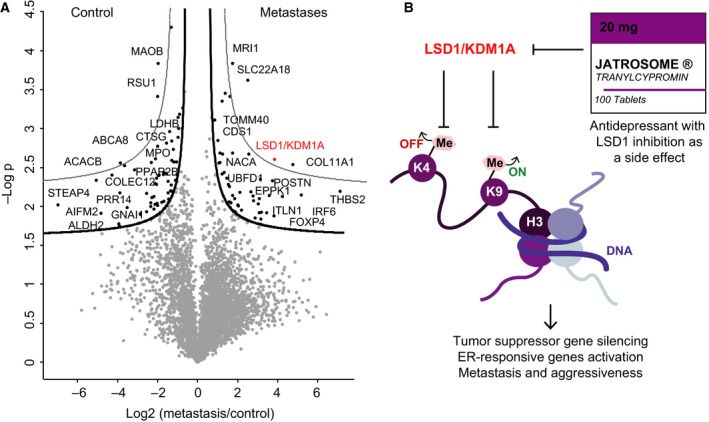
Proteins differentially expressed in the urachal carcinoma lung metastases. (A) Volcano plot of the p‐values (*y*‐axis) vs. the log2 protein abundance differences (*x*‐axis) between metastases and control, with lines of significance colored in black or gray lines corresponding to a 5% or 1% FDR, respectively. (B) Mechanisms of action of LSD1/KDM1A.

In an effort to derive therapeutic options, we first reduced the total number of significantly upregulated proteins by applying a more stringent cutoff (1% FDR). This yielded four significantly upregulated proteins in the metastatic tissue: MRI1, solute carrier family 22 member 18 (SLC22A18), collagen alpha‐1 (XI) chain (COL11A1), and lysine‐specific histone demethylase 1A (KDM1A, also known as LSD1) (Fig. 3A). Next, we asked which of these proteins were potentially druggable, which left us with LSD1 as the sole remaining candidate. We quantified LSD1 with 11 unique peptides, reaching an approximate sequence coverage of 20%, and found that it was 16‐fold more highly expressed in the metastases compared to the control.

LSD1 is an epigenetic regulator that demethylates both the activating histone mark H3K4me and the repressive mark H3K9me, thereby acting as a coactivator or corepressor, depending on cellular context. LSD1 has previously been reported as upregulated in multiple cancer types and its inhibition has antitumor activity in lung cancer (Mohammad and Kruger, 2016; Singh *et al*., 2015). These findings led to the development of multiple LSD1 inhibitors that are currently in clinical trials (Alsaqer *et al*., 2017; Mohammad and Kruger, 2016; Schmidt and McCafferty, 2007). Even though it was unclear whether the lung metastases would respond to an LSD1 inhibitor, there were no other rational or reasonable treatment options available at this point. However, extensive efforts to obtain one of these drugs for use in our patient ultimately proved unsuccessful. Fortunately, tranylcypromine a drug developed decades ago and FDA approved for the treatment of depression and anxiety (Burger and Yost, 1948) has recently been shown to irreversibly inhibit LSD1 as a side effect (Binda *et al*., 2010; Ulrich *et al*., 2017; Zheng *et al*., 2016). This analogue of amphetamine is a monoamine oxidase (MAO) inhibitor, an enzyme family that is mechanistically related to LSD1. Tranylcypromine and derivates of this drug already showed clinical efficacy for several conditions in clinical trials, including the treatment of acute myeloid leukemia. The local tumor board approved treatment with this drug, and our patient was prescribed a tyramine‐free diet, to prevent accumulation of tyramine (normally metabolized by MAO), which could lead to high blood pressure and culminate in a hypertensive crisis (Gillman, 2011; Ulrich *et al*., 2017). However, a baseline CT at the initiation of therapy revealed dramatic metastatic progression to the liver, concurrent with hepatic failure (Fig. S1D). The patient was then transferred to a palliative care ward and died soon after.

Mass spectrometry‐based proteomics is a multifaceted technology and further allowed us to investigate the plasma proteome of our patient. Based on our previously developed ‘plasma proteome profiling’ pipeline (Geyer *et al*., 2016a), we quantified more than 400 proteins in triplicate LC‐MS measurements, enabling quantification of inflammatory proteins, such as C‐reactive protein (CRP) and the majority of the complement system (Fig. S2). We identified the entire inflammatory panel which we have previously reported and found it to be clearly elevated compared to normal controls with CRP showing the strongest upregulation (>6‐fold) (Geyer *et al*., 2016b), reflecting the systemic inflammation commonly observed in patients with end‐stage malignancy and heavy metastatic load.

We also investigated whether the patient would be likely to respond to immunotherapy. MS‐based measurements did not reveal any expression of PD‐1 or PD‐L1 proteins, an observation that was later confirmed by immunohistochemistry, suggesting a poor response to immunotherapy‐based treatments (Fig. S3C,D). In addition, we did not observe any immune cell infiltration in the metastases.

### 2.4 Proteomic analysis of the primary tumor

**Figure 4:**
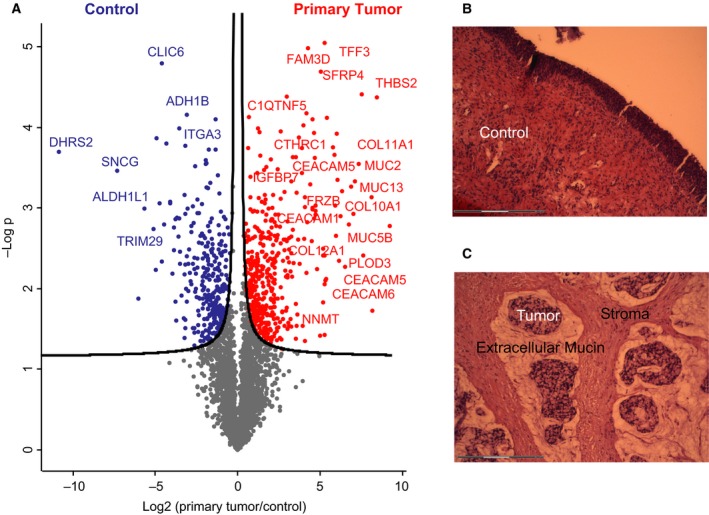
Differentially expressed proteins in the primary tumor. (A) Volcano plot of the p‐values vs. the log2 protein abundance differences between primary tumor and control, with significance lines (5% FDR) colored in black. H&E stainings of control healthy bladder wall (B) and the primary tumor (C).

To further investigate the proteomic landscape of our quantitative and in‐depth proteomic case study, we next analyzed the primary tumor, which had been preserved as FFPE material for several years. H&E staining showed that it was rich in extracellular mucin and stroma compared to healthy control tissue (Fig. 4B,C). Our proteomic analysis revealed major differences between the primary and healthy surrounding tissue (Fig. 4A). In total, we quantified approximately 4200 proteins and found that mucinous (e.g. MUC1 and MUC2) and mesenchymal proteins (such as THBS2, COL11A1, and CTHRC1) were significantly upregulated in the primary tumor compared to healthy surrounding tissue. Generally, the epithelial‐mesenchymal transition and thus mesenchymal gene upregulation are associated with poor prognosis in various malignancies including colorectal cancer and ovarian cancer (Chen *et al*., 2014; Rokavec *et al*., 2017; Sleeman and Thiery, 2011). The fact that mesenchymal proteins were highly enriched in the primary tumor is concordant with the later development of multiple and aggressive metastases. Interestingly, we also found that LSD1 appeared to be upregulated in the metastases compared to the primary tumor, albeit not significantly.

### 2.5 Next‐generation sequencing analysis of the metastases

To gain additional insights into the overall molecular mechanisms underlying urachal carcinoma, tumor etiology and to compare transcriptomics to proteomics, we also extracted RNA and DNA for subsequent next‐generation sequencing. The quality of the extracted RNA from the metastatic samples, however, was poor prohibiting direct transcriptomic analysis, which would have required additional steps in RNA stabilization. In contrast, we were able to isolate DNA of high quality, allowing us to perform exome sequencing on our samples. Comparing metastatic and surrounding tissues, we observed hundreds of mutations in coding regions, indicating a hypermutated phenotype, consistent with a previous report on urachal carcinoma (Kardos *et al*., 2017). Data were further filtered resulting in 320 high‐confidence somatic point mutations, including 83 exonic mutations (Methods and Table S1). In order to interpret the relevance of these mutations and also study the possible anticancer therapies, we further analyzed them using the cancer genome interpreter (CGI) (Tamborero *et al*., 2018). This tool helped us to annotate the tumorigenic potential of the called variants as well as to identify the alterations that could be therapeutically targeted and the observed response. CGI catalogue identified five proteins affecting driver mutations (one known mutation in COREAD: K117N in exon 4 of KRAS, and four predicted driver mutations [oncodriveMUT (Tamborero Noguera *et al*., 2016) E566V (MCC), D1046A (KDR), Y641C (FN1) and Q167 (TP53)]. Mutations in members of the EGFR pathway, including KRAS, are of particular interest as the use of EGFR inhibitors such as gefitinib has been described for urachal carcinoma recently (Collazo‐Lorduy *et al*., 2016; Singh *et al*., 2016; Sirintrapun *et al*., 2014). Unfortunately, several studies reported that patients with KRAS mutations in exons 2, 3, and 4 did not respond to EGFR‐targeted therapy (Bokemeyer *et al*., 2015; Douillard *et al*., 2013). We also found two intronic and two exonic somatic mutations of the LSD1 gene, which, however, scored neutral by mutation effect predictors (Fig. S4).

## 3 Discussion

Modern oncology is at a turning point where systemic cancer treatment is moving toward more personalized approaches based on molecular characterization of each individual's tumor (Schork, 2015). This is particularly promising for patients suffering from rare cancers, where standard chemotherapies often fail and large clinical studies are unlikely to be performed. In the near future, sequencing at the genomic, transcriptomic and proteomic levels might provide the basis for individual targeted treatment prescription and thereby change clinical practice. However, while genomics will always be the gold standard for identifying genome alteration in cancer, the spectrum of mutations by itself does not necessarily lead to clear therapeutic options, a problem that becomes even more acute when considering mutational heterogeneity of most tumors. These general challenges are well known and were reflected in our case study, where mutational analysis did not lead to a clear treatment recommendation. In contrast, our personalized MS‐based proteomic analysis performed robustly and quickly on both the lung metastases and the archived primary tumor. The significance of our study is its demonstration that proteomics has the potential to provide personalized therapeutic options for patients where standard clinical options have been exhausted.

The current standard treatment for localized urachal cancer is surgery, whereas chemotherapy is used in metastatic disease. Given the rarity of this cancer type, robust data from prospective trials on chemotherapy regimens are unlikely to be obtainable and evidence mainly consists of small retrospective cohorts. Due to the similarity of urachal cancer to colorectal adenocarcinoma and urothelial carcinomas, treatment regimens are generally extrapolated from these diseases, justifying the FOLFOX therapy prescribed to our patient. Targeted EGFR inhibitors (e.g. gefitinib) have been used for urachal carcinoma recently. To guide decisions concerning this alternative therapy option, we further looked into KRAS mutations and uncovered a missense mutation. However, EGFR‐targeted therapy was not initiated because patients with similar KRAS mutations as our patient did not respond to therapy (Bokemeyer *et al*., 2015; Douillard *et al*., 2013). Furthermore, the elevated liver enzymes of our patients were contraindications for such a therapy. In the search for possible further treatment options in this patient, we found that PD‐L1 and CD8 immunohistochemistry were also negative, suggesting a poor response to checkpoint inhibitors.

Lacking evidence‐based treatment options for our end‐stage patient, who was willing to exhaust all possibilities, we turned to MS‐based proteomic analysis, which identified LSD1 as a therapeutic target highly enriched in metastatic tissue. This promising treatment opportunity provided timely and actionable results to the patient and the clinicians. Of note, the proteomic sample preparation and data analysis were accomplished in only about 2 days, much faster than the genomic analysis. This highlights the promise of MS‐based proteomics in clinical routine, where fast target identification for cancer patients beyond standard treatment could be highly beneficial.

## 4 Conclusion

In summary, we demonstrated that fast and reproducible proteomics can create the possibility for clinicians to use proteomics for personalized diagnosis and treatment in the clinical setting. By combining genomic with proteomic data, we further informed therapeutic decisions. We aim to apply this workflow to cancer patients in a variety of chemorefractive tumors, in the hope of identifying additional treatment options for at least some of them.

## 5 Materials and methods

### 5.1 Sample preparation

Lung metastases were collected during surgery from the pleural cavity (pleura parietalis) and compared to surrounding noncancerous pleura tissue. In total, we analyzed six metastatic and six ‘control’ samples that originated from two metastases and surrounding tissue collected at different locations of the pleural cavity. Samples were washed three times with cold PBS before flash‐freezing the samples in liquid nitrogen and shipping on dry ice. The samples were split to enable genomic and proteomic analysis. The archived primary tumor was surrounded with healthy bladder tissue and preserved as FFPE material. The investigation was approved by the local ethical committee (2015‐540‐MA), and the experiments were undertaken with the understanding and written consent of the patient. The study methodologies conformed to the standards set by the Declaration of Helsinki.

#### 5.1.1 Proteomic sample preparation

Control and lung metastases samples were thawed on ice and prepared following the in stage tip sample preparation method with minor modifications (Kulak *et al*., 2014). Briefly, 100 μL of the reducing alkylating sodium deoxycholate buffer (PreOmics, Martinsried, Germany) was added to the samples before protein denaturation at 100 °C for 20 min. Samples were further homogenized by 15‐min sonication in a Biorupter (30 s on/off cycles, high settings). Proteins were then digested by Lys‐C and trypsin overnight at 37 °C and constant agitation. Peptides were acidified to a final concentration of 0.1% trifluoroacetic acid (TFA) for SDB–RPS binding and desalted before LC‐MS/MS analysis.

#### 5.1.2 Exome sequencing

DNA was extracted from tissues using the DNeasy Blood & Tissue Kit (Qiagen, Hamburg, Germany) according to the manufacturer's instruction. DNA quality was controlled by measurement of the 260/280 nm ratio using a NanoDrop photospectrometer and Exome Seq Libraries prepared from 100 ng of genomic DNA using the TruSeq Exome Library Prep Kit from Illumina. Briefly, the DNA was fragmented using a Covaris M220 and ligated to Illumina adapters. Exonic oligo probes were used to hybridize the coding exons and subsequently captured for enrichment of those targeted coding regions. The hybridization was performed overnight twice to ensure high specificity of the captured regions. The final libraries were checked on the Fragment Analyzer (AATI) and quantified by qPCR with KAPA Library Quantification Kit (Kapa Biosystems, Roche, Basel, Switzerland). Sequencing was performed with the Illumina NextSeq 500 using a mid‐output flowcell and a paired‐end mode 2x75 cycles, reaching 45 million reads per sample. The resulting reads for the tumor and normal samples were aligned to the hg19 reference genome with BWA (Li and Durbin, 2009) and afterward processed according to the GATK Best Practices recommendations for variant discovery (DePristo *et al*., 2011; McKenna *et al*., 2010; Van der Auwera *et al*., 2013). The discovered variant callset was then filtered and annotated using MuTect (Cibulskis *et al*., 2013), which allows identification of somatic point mutations with high confidence. We further interpreted the relevance of these mutations and possible anticancer therapies using the cancer genome interpreter (CGI) (Tamborero *et al*., 2018).

### 5.2 Liquid chromatography–MS analysis

Samples were measured on a quadrupole Orbitrap mass spectrometer (Kelstrup *et al*., 2014; Scheltema *et al*., 2014) (Q Exactive HF, Thermo Fisher Scientific, Rockford, IL, USA) coupled to an EASYnLC 1200 ultra‐high‐pressure system (Thermo Fisher Scientific) via a nanoelectrospray ion source. About 1 μg of peptides was loaded on a 40‐cm HPLC‐column (75 μm inner diameter; in‐house packed using ReproSil‐Pur C18‐AQ 1.9‐μm silica beads; Dr Maisch GmbH, Germany). Peptides were separated using a linear gradient from 3% to 23% B in 82 min and stepped up to 40% in 8 min at 350 nL per min where solvent A was 0.1% formic acid in water and solvent B was 80% acetonitrile and 0.1% formic acid in water. The total duration of the gradient was 100 min. Column temperature was kept at 60 °C by a Peltier element‐containing, in‐house developed oven. The mass spectrometer was operated in ‘top‐15’ data‐dependent mode, collecting MS spectra in the Orbitrap mass analyzer (60 000 resolution, 300–1650 m/z range) with an automatic gain control (AGC) target of 3E6 and a maximum ion injection time of 25 ms. The most intense ions from the full scan were isolated with a width of 1.4 m/z. Following higher‐energy collisional dissociation (HCD) with a normalized collision energy (NCE) of 27%, MS/MS spectra were collected in the Orbitrap (15 000 resolution) with an AGC target of 1E5 and a maximum ion injection time of 25 ms. Precursor dynamic exclusion was enabled with a duration of 20 s.

### 5.3 MS data analysis

Tandem mass spectra were searched against the 2015 Uniprot human databases (UP000005640_9606 and UP000005640_9606_additional) using maxquant version 1.5.3.34 with a 1% FDR at the peptide and protein level, peptides with a minimum length of seven amino acids with carbamidomethylation as a fixed modification and protein N‐terminal acetylation and methionine oxidations as variable modifications (Cox and Mann, 2008). Enzyme specificity was set as C‐terminal to arginine and lysine using trypsin as protease, and a maximum of two missed cleavages were allowed in the database search. The maximum initial mass tolerance for precursor and fragment ions was 4.5 ppm and 20 ppm, respectively. If applicable, peptide identifications by MS/MS were transferred between runs to minimize missing values for quantification with a 0.7 min window after retention time alignment. Label‐free quantification was performed with the MaxLFQ algorithm using a minimum ratio count of 1. All MS proteomic data have been deposited on ProteomeXchange via the PRIDE database with the data set identifier PXD008713.

### 5.4 Statistical analysis

Statistical and bioinformatics analysis was performed with the perseus software (Tyanova *et al*., 2016) (version 1.5.5.0), Microsoft Excel, and r statistical software. Proteins that were identified in the decoy reverse database or only by site modification were not considered for data analysis. Median of technical triplicates (referring to independent sample preparations) were calculated and mean log2 ratios of biological duplicates (two metastases and two control tissues collected at different locations of the pleural cavity), and the corresponding p‐values were visualized with volcano plots. We used *t*‐tests for binary comparisons and SAM with s0 = 0.1 and a FDR < 0.05 or <0.01 for the assessment of *t*‐test results in volcano plots. The FDR was corrected for multiple hypotheses based on permutation‐based FDR correction.

### 5.5 Histology and Immunohistochemistry

Tissue samples were fixed in 4% buffered formaldehyde, paraffin‐embedded, cut at 4 μm, and subjected to routine staining procedures including hematoxylin and eosin stain (H&E). Immunohistochemistry was performed using PD‐L1 monoclonal antibody clone E1L3N (Cell Signaling Technology, Frankfurt am Main, Germany) and diaminobenzidine (DAB) substrate chromogen detection system (Dako, Hamburg, Germany).

## Author contributions

SD acquired and interpreted the proteomics data, developed the concept, and wrote the manuscript. MCK interpreted the medical data and wrote the manuscript. ASD performed the genomic analysis, and MW helped with the DNA isolation. FC contributed to the data analysis. HMN performed the genomic measurements. SP investigated the histology of the tissues and did the immunohistochemistry stainings. PEG helped with the plasma proteome analysis. AM contributed to the interpretation of the data. PN developed the concept, supervised the study, and edited the manuscript. MM designed and supervised the study and edited the manuscript. All the authors have approved the final version.

## Supporting information


**Fig. S1.** Sectional imaging.
**Fig. S2.** Plasma proteome.
**Fig. S3.** H&E stainings.
**Fig. S4.** Mutation diagram of LSD1.

**Table S1.** Somatic point mutations.
