# Supplementary material for: Rapid proteomic analysis for solid tumors reveals LSD1 as a drug target in an end‐stage cancer patient
Source: Mol Oncol. 2018 Jun 14;12(8):1296–307. doi: 10.1002/1878-0261.12326 (PMC6068348; doi:10.1002/1878-0261.12326)
Supplement: Supplementary file 1 — Fig. S1. Sectional imaging. Fig. S2. Plasma proteome. Fig. S3. H&E stainings. Fig. S4. Mutation diagram of LSD1. [file MOL2-12-1296-s001.pdf]

Supplementary Figures

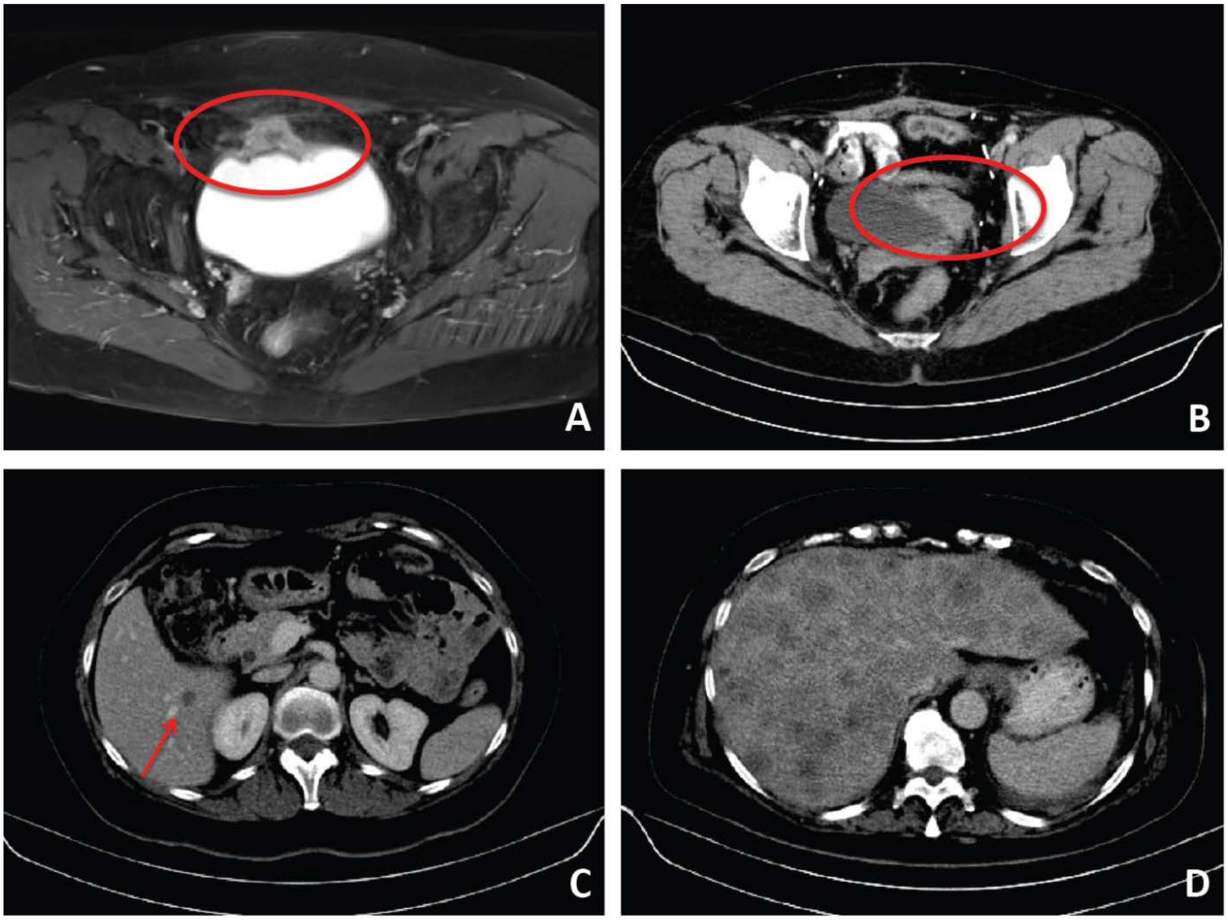

Supplementary Figure 1 Sectional imaging A) MR-imaging of the primary tumor showed a suspicious mass at the anterior bladder wall (red circle). B-C) Follow-up CT-imaging revealed local recurrence nine months after partial nephrectomy (red circle) and a hepatic metastasis (red arrow). D) Massive progression of hepatic metastases in the CT scan prior to LSD1 therapy.

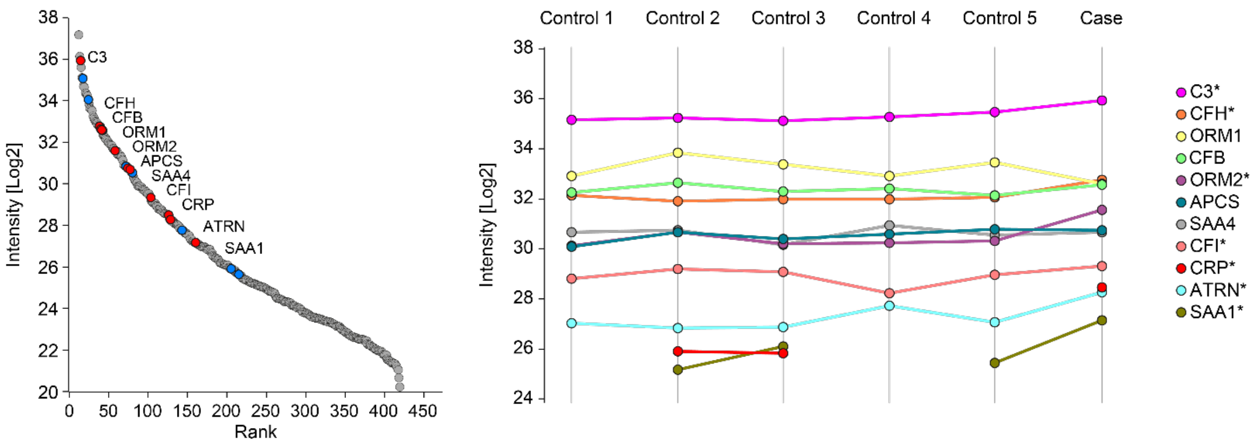

Supplementary Figure 2: Plasma proteome abundance rank of the patient and five control samples derived from entirely healthy individuals. The previously reported inflammatory panel and other complement proteins are highlighted in red and blue.

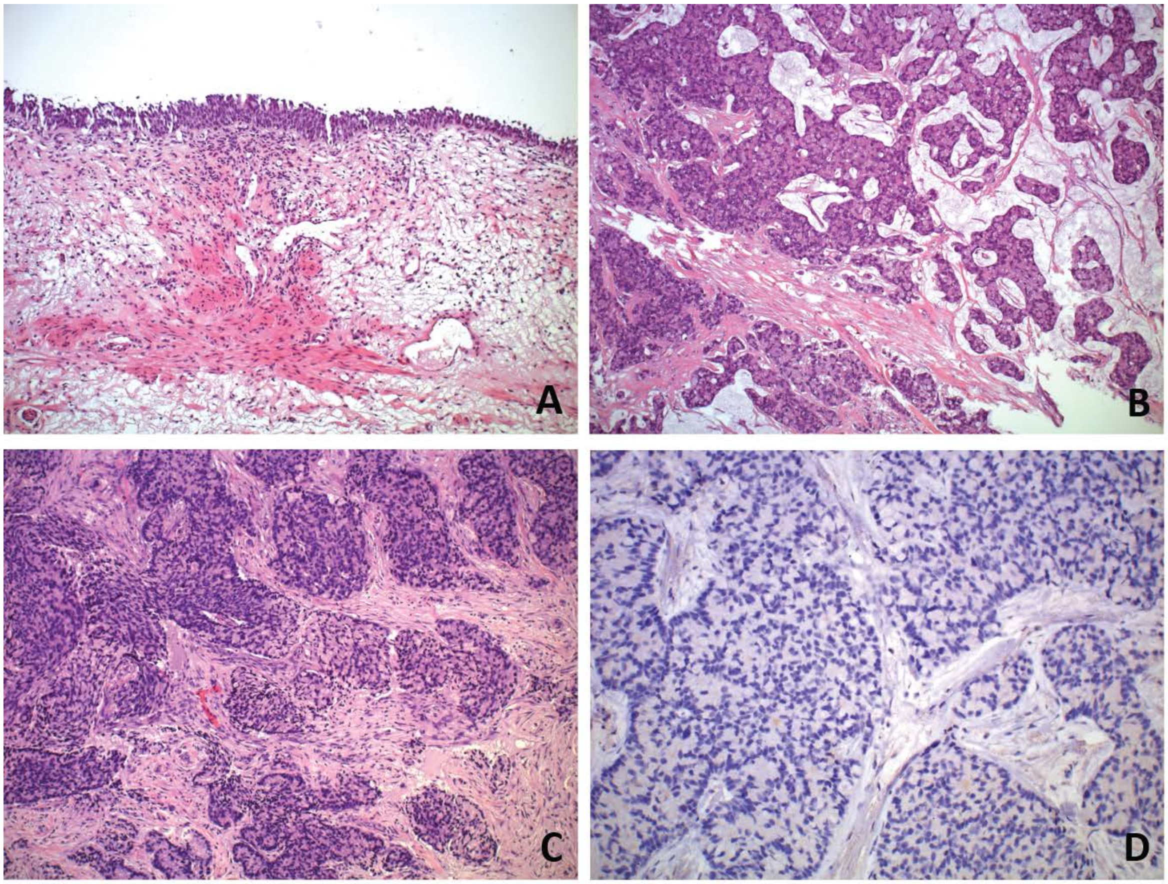

Supplementary Figure 3: H&E stainings. A) Healthy urothelium of the partial cystectomy specimen. B) Primary mucinous urachal adenocarcinoma. C-D) Hepatic metastasis of the urachal carcinoma with negative PD-L1 immunohistochemistry.

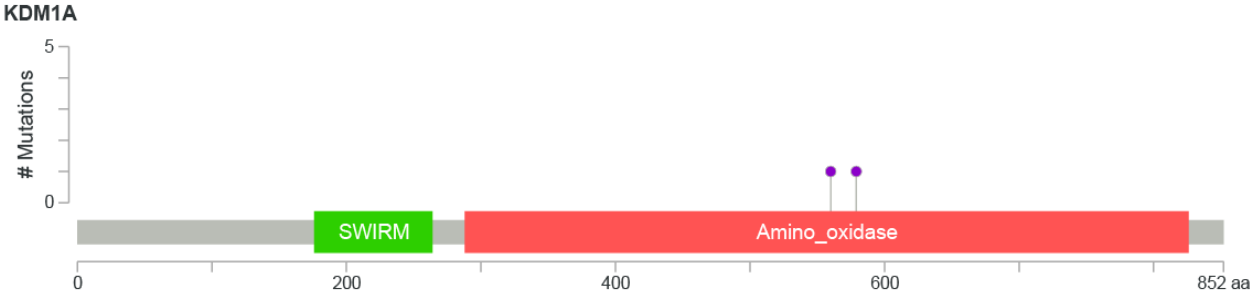

Supplementary Figure 4: Mutation diagram of LSD1.
